# A Review: The Effect of Bovine Colostrum on Immunity in People of All Ages

**DOI:** 10.3390/nu16132007

**Published:** 2024-06-25

**Authors:** Hao Duan, Qian Sun, Chao Chen, Rongchang Wang, Wenjie Yan

**Affiliations:** 1 College of Biochemical Engineering, Beijing Union University, Beijing 100023, China; dhuanao@163.com; 2 Beijing Key Laboratory of Bioactive Substances and Functional Food, Beijing Union University, Beijing 100023, China; 3 Native Nutrition and Medical Research Institute, Tianmeijian Biotechnology (Beijing) Co., Ltd., Beijing 100101, China; 4 Research and Development Center, Jiangsu Tianmeijian Nature Bioengineering Co., Ltd., Nanjing 210038, China; 5 Research and Development Center, Nanjing Daily Nutrition Biotechnology Co., Ltd., Nanjing 211215, China

**Keywords:** bovine colostrum, nutritional composition, adolescents, adults, elderly, immunity

## Abstract

Bovine colostrum provides newborn calves with strong passive immunity, which will further affect the immunity of their offspring. Compared with other commercial dairy products, bovine colostrum emphasizes the limit of aflatoxin M1, pathogenic bacteria, microorganisms, antibiotics, stimulants, and other items, so it is safe to use. There are many reports that the use of bovine colostrum as a breast milk fortifier for preterm infants provides necessary immune support for premature infants, but the selection of bovine colostrum products chosen must be free of Bacillus cereus because they are very dangerous for premature infants. This also emphasizes that for the bovine colostrum that is used in preterm infants, more clinical research support is needed. At the same time, it should also be emphasized that the composition of BC is different from that of human colostrum, in particular, the main protein of BC is casein, while the main protein in breast milk is whey protein, especially α-lactalbumin, which together with ovalbumin is still the reference protein with the best biological value, especially for muscles. Therefore, bovine colostrum is currently not a complete substitute for breast milk. In recent years, in addition to reports of bovine colostrum use in preterm infants, studies have also found that bovine colostrum has immunomodulatory and promoting effects in adolescents, adults, and the elderly. This suggests that bovine colostrum has the potential to provide appropriate immune support for people of all ages. Therefore, this study aimed to evaluate the quality of nutritional characteristics of bovine colostrum on three dimensions. The effects of bovine colostrum on people of all ages is a narrative review of the effects of bovine colostrum on immunity in people of all ages. This review identified several classes of immunoactive substances in bovine colostrum, including immunoglobulins, cytokines, and enzymes, and compared the nutritional composition of bovine colostrum with mature milk, colostrum and mature milk in full-term breast milk, and colostrum and mature milk in preterm breast milk, to demonstrate that bovine colostrum provides a rich range of immunoactive components. In addition, the influencing factors affecting the quality of bovine colostrum (immunoglobulin) were reviewed, and it was found that individual differences, environmental factors, and processing methods had a great impact on the quality of BC. More importantly, the immunomodulatory effects of bovine colostrum in people of all ages were reviewed in detail (with an emphasis on preterm infants and immunocompromised children in neonates) as evidence to support the immunity effects of colostrum in people of all ages. This review hopes to use the above evidence to make people understand the health role of bovine colostrum as having a human immunomodulatory effect, and at the same time, when seeing the potential value of bovine colostrum in the future, the limitations of its application should also be deeply re-explored, such as lactose intolerance, allergies, etc., to provide effective solutions for the wide application of bovine colostrum.

## 1. Introduction

Bovine colostrum (BC) is an oral immune-boosting agent that has been popular for thousands of years, and has been widely used as an antimicrobial and antiviral agent since before the advent of penicillin and antibiotics [1]. Some earlier studies defined BC as six milk cycles in the 3~4 days before farrowing, but now it is generally correct to refer to it as milk expressed within 72 h of farrowing from healthy, normally reared cows that are free of infectious diseases and mastitis [2]. Newborn calves receive passive immunity from their mothers through ingesting colostrum, which provides the newborn with essential nutritional support for energy, immunity, growth, and development. A large number of the active ingredients in BC have a cross-species effect, which can replace (or partially replace) colostrum/milk supplementation for other mammalian neonates. BC is generally safe for consumption. When 10% BC was added to the rat diet, after 90 days of feeding, the rats did not show any abnormalities in physiological, physical, and tissue lesions [1], so it is suitable to provide necessary nutritional support and immunity for newborns [3]. At present, multidisciplinary research evidence has confirmed that BC is rich in nutrients and a large number of immune factors and growth factors, such as immunoglobulins, lactoferrin, lysozyme, insulin-like growth factors, epidermal growth factors, etc., having very clear health benefits for immune regulation, gastrointestinal tract improvement, growth and development, ageing, antibacterial, and anti-inflammatory functions [4]. Evidence that colostrum promotes immune construction can be determined by comparing the compositional differences between colostrum and mature milk. Both human colostrum and bovine colostrum have been found to contain a large amount of protein, fat, growth factor cytokines, and a small amount of lactose compared to mature milk [5], and these rich immunoactivity components largely determine the survival rate of newborns. Studies have shown that newborns can absorb macromolecules such as immunoglobulins through the gastrointestinal tract in the first few days to obtain passive immunity. When the gastrointestinal tract matures, this convenient route becomes difficult. Instead, infants influence the microbial distribution in the large intestine through dietary components, enhance intestinal protection against pathogens and toxins, and stimulate a systemic immune response to antigens [1]. However, it has been shown that the content of immunoactivity proteins in mature milk decreased somewhat, and high concentrations of lactose were negatively correlated with casein content while regulating milk yield [5,6]. Therefore, it is necessary to compare and analyze the nutritional characteristics of bovine colostrum, bovine mature milk, normal breast milk, and breast milk of preterm infants, to further analyze the reasons and advantages of using bovine colostrum for improving immune activity. Furthermore, the influencing factors affecting the quality of bovine colostrum (immunoglobulin) were reviewed. At the same time, this review also provides a detailed review of the effect of bovine colostrum on the immunity of people of all ages, as evidence to support the immunity effects of colostrum in people of all ages. Therefore, this paper aims to develop a comprehensive study investigating three dimensions (nutritional properties; influencing factors of bovine colostrum quality; the effect of bovine colostrum on people of all ages). This is a narrative review of the role of bovine colostrum on immunity in people of all ages.

## 2. Method

Narrative examination is conducted in three steps: conducting a search, examining the abstract and full text, and discussing the results. To this end, PubMed, Scopus, Science Direct, Web of Science, Science Direct, CNKI, and Google Scholar databases were searched to identify relevant studies as the review developed. The final search was conducted in January 2024 and included international English-based articles, online reports, and e-books. The keyword “bovine colostrum” was used in conjunction with other terms such as immunity, nutrition, influencing factors, infant, neonatal, juvenile, child, elderly, adult. After completing the search, the abstracts were read to make sure they related to the topics of interest. All duplicates were removed, and abstracts of the remaining articles were reviewed to ensure they met the review inclusion criteria. The criteria for eligibility are the three dimensions (nutritional profile; influencing factors of bovine colostrum quality; the effects of bovine colostrum on people of all ages). Therefore, studies of interest focusing on these three dimensions were summarized and synthesized to integrate narrative reviews. As this is a narrative review, it was not necessary to record a literature search on a specific platform.

## 3. The Main Nutritional Composition of Bovine Colostrum

BC is usually used as a human milk fortifier due to its high nutritional value, but its nutritional composition is greatly influenced by individual differences in cows [7]. Table 1 summarizes the differences between the nutritional composition of BC and mature milk, colostrum and mature milk of full-term breast milk, and colostrum and mature milk of preterm breast milk. It is not difficult to see that BC has higher energy, fat, protein, casein, whey, immunoglobulin, and IgG than several other milk sources. These components support BC to regulate or promote immunity in offspring. The contents of IgA, lactose, lactoferrin, and lysozyme in BC were significantly lower than those in human milk. Interestingly, the protein content in the breast milk of preterm infants was significantly higher than that in the breast milk of term infants, suggesting that preterm infants have higher nutrient requirements, and that it is important to provide the offspring with the necessary nutrients to support the rapid construction of immunity through breastfeeding. Studies have shown that infants obtain basic nutrients such as amino acids, glucose, and fatty acids through the placenta in the mother’s body. In particular, long-chain polyunsaturated fatty acids (LCPUFA), docosahexaenoic acid (DHA), and arachidonic acid (ARA), which are not found in high levels in colostrum, and essential for brain development. However, because some vitamins cannot break through the placental barrier, this means that newborns need to quickly obtain a large number of more complex and abundant nutrients such as protein, lactose, growth factors, and immune factors through breastfeeding to obtain innate immunity until the adaptive immunity related to the specific environment matures [3,8,9]. In particular, breastfeeding combined with appropriate nutritional supplements helps preterm babies to acquire a strong immune system. However, BC is rich in immunoactivity components, and the nutritional composition of BC is relatively close to that of human milk, so it is often used as a breast milk fortifier for preterm infants. As a breast milk supplement, BC not only enhances the antimicrobial activity of human milk but also promotes the improvement of bowel habits in preterm infants [10,11]. In addition, oral BC reduced feeding intolerance, necrotizing enterocolitis (NEC), late-onset septicemia, and mortality in preterm infants [12,13,14], suggesting that BC may have some immunomodulatory role. Human milk itself, HMO, and microbiota furnished by the mother are proven to have an immunological effect and protect intestinal cells and NEC, mortality and brain development. Moreover, genetic and epigenetic programing modifies the composition of breast milk of the human mother. So, BC does not offer human specific changes and protection. With the advancement of lactation, most nutrients in mature milk decrease, and lactose content increases, which is conducive to regulating milk production [5,6,15]. In addition, BC has a high concentration of trace elements, such as vitamins and minerals, which are also involved in various physiological processes in the body, including defense, immunity, lipid digestion and absorption, and antioxidants [16]. At the same time, the issue to be noted is that the protein content of BC is too much higher vs BM and MPM. So, a higher protein intake is necessary for growth but does not need to be higher than 4 g/kg/day in the early preterm nutrition. Therefore, the use of colostrum in preterm infants should be based on the amount of intake. In addition, the casein content in BC is higher than in BM, which may reduce gastric emptying and digestibility in preterm infants, so care needs to be taken when choosing a colostrum product that does not contain too much casein.

## 4. Immunoactive Substances in Bovine Colostrum

The immunoactive substances with immune activity in BC are mainly immunoglobulins (Ig), which are very abundant. At the same time, Ig and antibacterial components such as lactoferrin, cytokines, lactoperoxidase, and lysozyme play a very important role in the body’s immunity.

### 4.1. Immunoglobulins

Immunoglobulins (Igs) are a class of globulins with antibody activity and similar chemical structure to antibodies, which can bind to antigens to reduce the toxicity of antigens, and at the same time, they can also bind to complement to resist the invasion of exogenous adverse pathogens and improve the body’s immunity [36]. Igs are derived from plasma/B cells and are highly specific and capable of targeted destruction of appropriate pathogens to provide the body with necessary immune support [37]. The high content of immunoglobulins in BC mainly includes IgG, IgA, and IgM, in addition to IgE and IgD. Among these, IgG is the main substance of total immunoglobulins in BC, with a content of up to 80~85% [17], followed by IgA and IgM, so IgG is often used as an important indicator to evaluate the quality of BC. In humans, immunoglobulins can cross the placental barrier to provide essential nutritional support to the fetus, whereas in calves, Ig can only be obtained through lactation. The high concentration of Ig in BC suggests an important role in immune acquisition, as strong metastases of IgA, IgM, and IgG, especially IgG, have been observed during elevated inflammation in the birth canal [38].

### 4.2. Cytokines

BC contains considerable cytokines, such as epidermal growth factor (EGF), transforming growth factor (TGF), insulin-like growth factor (IGF), vascular endothelial growth factor (VEGF), etc. [34], which are produced by a variety of cells, especially immune cells, and therefore have powerful immunomodulatory effects [39]. Among them, EGF is a peptide composed of 53 amino acid residues and 3 intramolecular disulfide bonds, which plays an important role in regulating cell growth, survival, apoptosis, proliferation, and differentiation, and has a strong protective effect on the integrity and homeostasis of the intestinal barrier, so it is very important for immunity and intestinal health [40]. TGF-β is a potent regulatory cytokine that plays a role in the immune system by regulating the proliferation, differentiation, and survival of lymphocytes to maintain tolerance. Second, the main mechanism by which TGF-β can control the initiation and regression of inflammatory responses is accomplished by modulating the chemotaxis of lymphocytes, natural killer cells, macrophages, dendritic cells, mast cells, and granulocytes [41]. These cytokines provide the body with the immunomodulatory effects it wants.

### 4.3. Enzymes

The antimicrobial and bactericidal components in BC are derived from lysozyme, lactoperoxidase, and lactoferrin [42]. Lysozyme is a conserved antimicrobial protein that is a non-specific immune factor that can participate in a variety of immune responses in the body [43]. Lysozyme exerts its antimicrobial effect by hydrolyzing and killing bacteria through cell wall peptidoglycan [44]. The number of cytokines and lysozyme in BC was significantly higher than that in mature milk, which again reflected the urgent need for immunity in newborn calves. Although the current data show that the number of cytokines and lysozyme in BC is lower than that in human milk, they have important physiological anti-infection, antibacterial, and immunomodulatory effects.

Lactoperoxidase has broad substrate specificity and is thermotolerant to act against different microorganisms [45]. Bovine colostrum contains a certain amount of lactoferrin, but the content drops rapidly after experiencing high temperatures, especially pasteurization. Lactoferrin is a natural glycoprotein with a variety of physiological effects such as antibacterial, immunomodulatory, antioxidant, anti-inflammatory, and anticancer. In addition, lactoferrin has a high safety profile and high oral bioavailability [46]. Therefore, it is very suitable as a dietary supplement to provide effective protection for human health.

### 4.4. Other Proteins

As can be seen from Table 1, BC contains a higher concentration of protein, which is five times higher than that of mature milk, and its content is higher than that of healthy breast milk and breast milk of preterm infants, which may be due to the higher levels of casein and immunoglobulin, but it may also be due to the failure of the detection methods used in the literature. In addition, the protein content of bovine colostrum drops dramatically after high temperature pasteurization, which explains the wide range of data in Table 1. At the same time, the casein content in BC is higher than that in BM, which will reduce the gastric emptying and digestibility of premature infants, which suggests that when choosing colostrum products, we should pay attention to the selection of their ingredient list, and the dosage should be used in strict accordance with the doctor’s advice, so that it is not easy to overdose. BC protein can be divided into whey protein and casein, both of which have good antioxidant activity and immune activity [47]. Casein is the main protein, but it will decline rapidly after high temperature sterilization, so the processing technology of bovine colostrum should be studied in detail in the future [17]. Casein is a high-quality source of protein, and it is found in cow’s milk in the form of colloidal casein micelles. Studies have shown that the casein micelles in BC are larger in diameter, and with the increase in lactation, the casein micelles are significantly reduced in diameter and content, reflecting the characteristic nutrient requirements and metabolic characteristics of the neonatal offspring [48]. This is because it has been reported that neonates can absorb macromolecules such as immunoglobulins through the gastrointestinal tract during the first few days to acquire passive immunity, and then this ability gradually decreases [1]. As a result, newborns have the strongest immunity on the first day and a decrease in immunity from day 0 to 6 months. Whey protein mainly contains a variety of active ingredients such as α-lactalbumin, immunoglobulin, growth factors, lactoferrin, etc., and its essential amino acids (including branched-chain amino acids, containing about 26%) [49,50] and whey protein contain a variety of active peptides that are released after enzymatic hydrolysis to promote the body’s health needs [51]. Casein and whey protein primarily provide muscle development and energy to the body. It is very important for the body’s immunity.

## 5. The Main Factors Affecting the Quality of Bovine Colostrum

The quality of BC is affected by a combination of factors, and its nutritional composition is also affected to a certain extent, which mainly comes from individual differences, environmental differences, and subsequent processing, so some of the statistical data in Table 1 have a large range of values. At present, the concentration of IgG in BC is mainly used as a key indicator to evaluate the quality of BC, and the evaluation criterion is that the IgG content in BC should be ≥50 g/L, and the higher the content, the higher the quality [52]. However, the evaluation index will be adjusted accordingly with different application scenarios, so when evaluating the quality of BC, we should not only focus on the change in IgG content but also combine the target product. Therefore, it is necessary to summarize and sort out the factors affecting the nutritional composition of BC, to provide a certain reference for obtaining high-quality BC in the future.

### 5.1. Individual Differences

Individual differences are complex, including parity, prenatal nutrition, colostrum quality, and other factors. The changes in IgG concentrations were mainly related to the number of colostrum, parity, and time from delivery to colostrum collection [53]. The quality of colostrum in high-yielding cows is significantly higher than that in primiparous cows [54]. In a study that collected colostrum over one year from a dairy farm and evaluated the effects of multiple factors on BC quality (evaluated using immunoglobulin concentration), the quality of colostrum improved with parity and dry milk duration and decreased with colostrum quantity, humidity, and humidity index ≥ 72 h, and the factors affecting colostrum quality were colostrum quantity > parity > length of drought period > climatic factors [55]. In another study, it was also confirmed that the IgG content of BC collected at 2 h postpartum was as high as 113 g/L, which was significantly higher than that of BC collected after 6 h postpartum, and that the concentration of IgG in colostrum was as high as 132 g/L in the third and above cows at 2 h postpartum, which was significantly higher than that of cows lactating for the first or second time (≤100 g/L) [56]. This suggests that the collection of BC immediately after postpartum helps to obtain higher levels of IgG and also contributes to the successful passive immunity of calves. The increase in IgG with parity may be due to the higher level of immune system exposure in high-parity cows, followed by a greater increase in the density of BC after the third party, which may also lead to an increase in IgG concentrations in BC [52]. In addition, the effect of individual factors on Ig is also manifested in the length of drought periods [57], but this effect is controversial in different studies, indicating that BC is affected by individual factors in a variety of ways. The influence of individual differences on BC quality may also be related to breed differences, but this factor is further influenced by various factors such as genetics, environment, and management [58], so it is rarely discussed in current studies.

Prenatal nutrition is also an important factor affecting the quality of BC, and some studies have found that oral administration of 150 g/d of organically modified clinoptilolite zeolite before prenatal is beneficial to improve the quality of colostrum in Holstein primiparous dairy cows, especially the concentration of IgG in it, and has a good safety profile [59]. By supplementing with 100 kg·per day starting 5 weeks before childbirth, the BW dose of moringa oleifera leaf powder helps to increase the content of fat, protein, lactose, and total solids in BC, and can significantly increase the IgG content in BC, which has a certain effect on the immunity of cows and newborn calves [60]. In addition, prenatal supplementation with a dose of 48 g/d nicotinamide was found to increase the concentration of IgG in BC by 1.36% [61].

In addition to parity, prenatal nutrition, and the amount of colostrum mentioned above which are important factors for individual differences in the quality of BC, factors such as the health status, breed, and dry milk period of dairy cows will have a certain impact on the quality of BC [15].

### 5.2. Hereditary Factor

Among the environmental factors, season is also one of the factors that affects the quality and yield in BC. Two studies in Moscow and Italy found that cows calving between August and November had higher levels of Ig in BC [62,63]. It is hypothesized that this may be due to photogenesis factors, as circulating prolactin concentrations are consistently influenced by changes in the photocycles [64], and these hormones further affect milk production. Because light exposure inhibits the release of melatonin, it results in low levels of melatonin circulating during the day and significantly higher levels at night, affecting levels of hormones such as prolactin and IGF-1, which in turn can cause differences in milk production [65]. Overall, prolonged light cycles contribute to increased milk production [66,67]. However, photoperiod does not appear to be significantly associated with IgA and lactoferrin levels [68].

### 5.3. Storage and Processing Methods

Most of the immunoreactive components in BC are proteins, so heat treatment and processing can easily cause their content loss, which affects the quality and immune activity of BC. Raw bovine colostrum has certain infectious pathogens, which are susceptible to microbial hazards, and exogenous microorganisms will bind to IgG to prevent the body from absorbing IgG. Pathogens may also adhere to intestinal cells, increasing intestinal permeability, which leads to the susceptibility of newborn calves which ingest the colostrum to microbial infection and death [69]. Further, the use of heat treatment is beneficial to kill microorganisms in colostrum and prolongs its shelf life, but this operation also affects the reduction in immunoreactive components in BC. Heat treatment of BC at 60 °C for 60 min or more results in decreased immunoglobulin, casein, and lactoferrin content in colostrum [70,71], but the reason for this loss may be related to prolonged heat treatment. This is demonstrated in two other studies, as treatment at 60 °C for 30 min had no significant effect on IgG in BC [71,72]. In addition, compared with the changes in bioactive components in colostrum treated with raw colostrum, standard pasteurization (72 °C, 15 s), mild pasteurization (63 °C, 30 min), and gamma irradiation (14 kGy dose) heat treatment reduced the levels of bacteria and IgG in BC, but the combination of mild pasteurization and gamma irradiation increased the antimicrobial and biological activity of BC compared with standard pasteurization [73]. Most current studies support the positive effect of heat treatment of bovine colostrum at ≤60 °C or the standard low-temperature approach, and that early administration of heat-treated BC is beneficial for the transfer of passive immunity [74]. However, it should be noted that pasteurization cannot destroy the spores of Bacillus subtilis. So, in addition to this sterilization method, the processing of bovine colostrum may also involve the disinfection of ultraviolet or ozone; the regular disinfection of production equipment; the necessary pretreatment and testing of raw colostrum before entering the assembly line; and the most important thing is that the processing of bovine colostrum from raw materials to finished products is to be always kept at a low temperature. Through filtration, freeze-drying, and irradiation sterilization, it can remove and inhibit bacillus microorganisms to the greatest extent. After testing, there is no microbial contamination, even if a very small amount of bacillus remains, it does not have the conditions for spore germination and will not cause microbial contamination, so better improves the safety of bovine colostrum.

In summary, combined with the existing research, the disinfection method at a low temperature helps retain a large number of active ingredients in BC, and this method is also conducive to avoiding adverse physical effects such as BC agglomeration and odor.

### 5.4. Other Factors

Feeding management may also lead to changes in the quality of BC, and it has been found that dairy cows vaccinated at 28 days of expected farrowing and started on an acid-producing diet 21 days before farrowing can increase the concentration of IgG in BC [75]. In addition, genetic traits also have a role in the quality of BC, but this is controversial and has had opposed results in different studies [62,76,77,78]. Again, this reflects the complex diversity of factors influencing BC quality.

## 6. Supporting Evidence for Immunity in People of All Ages with Bovine Colostrum

**Figure 1:**
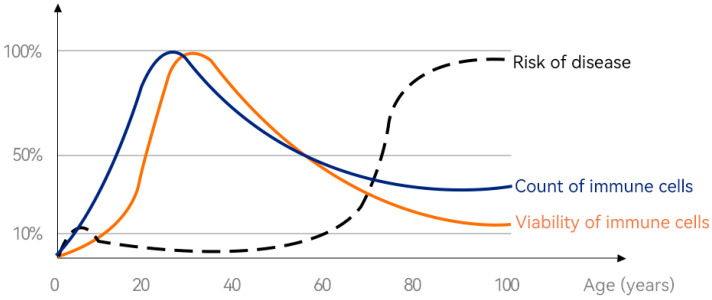
Changes in immune cell viability from “birth” to “lifetime”. (Immune cells’ senescence causes the body to age, and the key to anti-ageing lies in the vitality and number of immune cells. Figure courtesy of [79]).

Figure 1 illustrates changes in immune cell viability and immune cell numbers throughout the human life cycle. It can be seen that at the age of 0~30 years old, the number of immune cells and the activity of immune cells in the body are on the rise, and the immunity at this stage is gradually consolidated and improved with the increase in age. However, after the age of 30, the number of immune cells and the activity of immune cells in the body gradually decline, and the body’s immunity also declines. Correspondingly, the incidence of some diseases has also increased. Therefore, effective nutritional strategies should be proposed to improve our immunity and reduce the risk of disease. Bovine colostrum is rich in immunoreactive ingredients that can be absorbed by the body by oral administration and bring effective immune-boosting effects. The following is a detailed review of the immune-promoting effect of bovine colostrum on the whole life cycle of humans, and Table 2 summarizes the immunomodulatory or immune-promoting effects of BC in the population at each stage.

### 6.1. Colostrum Contributes to the Rapid Construction of Immunity in Preterm Infants

**Figure 2:**
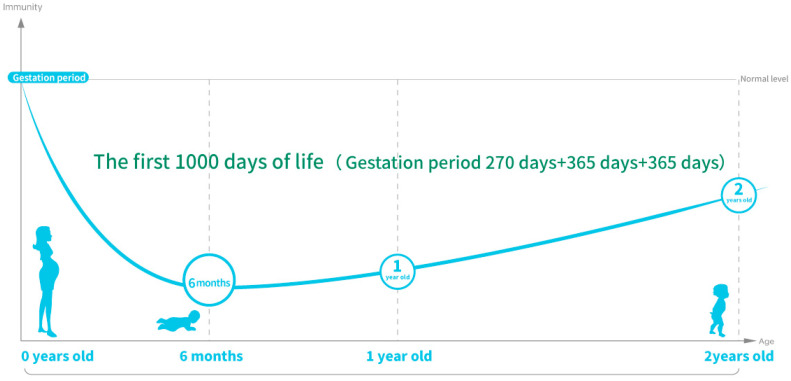
Changes in immunity in the first 100 days of life. (Because the maternal placenta and breast milk can provide the baby with immunoglobulins, there is little illness before 6 months; after 6 months, the immunoglobulin is at its lowest point, and the autoimmune globulin level is still very low, so supplementing with oral active immunoglobulin is key; figure drawing is based on [79,80,81,100]).

Mammalian newborns are unable to chew and digest solid food, so their only way to survive is through breastfeeding. In general, infants have poor immunity at 0~6 months of age, because they have not yet established a complete immune system, so the demand for nutrients is high, and the immune system begins to gradually improve after 6 months [80,81], and the food exposed to by the mother during pregnancy will affect the mother’s intestinal flora and further determine the adaptive immunity of the newborn [82]. Figure 2 shows the changes in immunity in the first 1000 days of life, and it is not difficult to see that the immunity of infants before 6 months of age is very fragile, which will lead to the infants being very dependent on breast milk for immune and nutritional support. Preterm infants, especially very preterm infants under 32 weeks gestation, have higher nutritional requirements for growth and development. High concentrations of immune factors can be detected in the colostrum of mothers of preterm infants [83], which once again confirms that breast milk is an important source of nutrition for preterm infants, and the immune components in it may provide the necessary immune support for infants. Neonates and premature infants have low immunity, incomplete intestinal function, and are susceptible to external viruses and bacteria, resulting in a high incidence of necrotizing enterocolitis (NEC), late-onset sepsis (LOS), and viral infection. Breastfeeding has been reported to help reduce the incidence of feeding intolerance, NEC, and LOS [84,85,86], but the milk supply of mothers of preterm infants is often insufficient to meet the nutritional and immune needs of preterm infants. At the same time, there are large individual differences in the nutrient composition of breast milk, and there are large differences throughout the lactation cycle [87,88]. Therefore, it is necessary to fortify the infant with additional nutritional fortifiers in addition to breast milk intake [89]. BC has a high content of immunoglobulin G (IgG), which is an important component of the body’s immune system, and newborn calves receive high levels of IgG and passive immunity from cows by supplementing with bovine colostrum within two hours of birth [56,90]. BC is rich in growth factors, cytokines, lactoferrin, lysozyme, and other components that can be absorbed through the intestine through oral administration to promote the rapid acquisition of immunity in newborn calves [91,92]. Bovine colostrum has been found to enhance the antimicrobial activity of breast milk in previous studies [10] and can be used as a breast milk supplement to feed preterm infants [33]. T regulatory cells (Tregs) have a modulatory effect on the immune system [93], and infants with NEC and LOS generally have the characteristics of low abundance of Tregs [94,95], and BC supplementation can help increase Treg levels and reduce the severity of sepsis and mortality in preterm infants, although the incidence of NEC and LOS in the BC-fed group was not statistically significant compared with that in the control group in this experimental study [96], suggesting that the dose of intervention for BC should be considered in future studies. In animal studies, human milk enhancers with BC have been found to increase intestinal protein intake in preterm infants and promote intestinal maturation and immunity in preterm infants [97]. In addition, BC has been shown to have a passive immune effect in children with anti-human rotavirus antibodies [98]. Dietary intervention with BC containing human rotavirus antibody in gastroenteritis mice and normal infants significantly improved the passive protection of managed mice against human rotavirus infection, and compared with the control group, the BC group did not have a single case of rotavirus-induced diarrhea, while 70% of the infants in the control group had diarrhea symptoms. Interestingly, this study also found that IgG and IgA purified from bovine colostrum containing human rotavirus antibodies did not protect against rotavirus infection, suggesting that other immunoreactive components other than immunoglobulins in BC may be involved in immune protection [99].

For example, RHB-602-2005 “Bovine colostrum powder” issued by the China Dairy Industry Association highlights aflatoxin M1 (≤5.0 μg/kg), pathogenic bacteria (intestinal pathogenic bacteria and pathogenic cocci, which are not detected), nitrate (NaNO_3_, ≤100 mg/kg) and nitrite (NaNO_2_, ≤2 mg/kg), and other ingredients, some brand companies will also focus on testing 60 antibiotics and 248 stimulants for bovine colostrum to ensure the high safety of products for preterm infants. Current research has also shown that BC as a breast milk supplement for preterm infants may help them quickly obtain the nourishment of immunoactive ingredients, thereby promoting the gradual improvement of immune system construction, to reduce the incidence of various diseases in infancy and have a high safety profile. However, the current number of clinical studies is insufficient to fully support the significant reduction of NEC and LOS in infants with BC, and the results of related studies are also controversial. In short, for special populations such as infants and young children, more attention is paid to the intake dose and intake cycle of bovine colostrum, and the dosage of bovine colostrum is adjusted according to the regular testing, which provides better immune support.

### 6.2. Bovine Colostrum Helps to Strengthen the Immunity of Adolescents and Children

Children and adolescents are at an important stage of development, and it is also a critical stage for strengthening and improving immunity. Malnutrition, as well as bacterial gastrointestinal and respiratory tract infections, have become serious public health problems, which have led to a variety of adverse events, such as stunting or retardation and increased mortality, which are closely related to immunocompromise [101]. Bovine colostrum provides natural growth stimulators, such as islet growth factor (IGF), which can promote intestinal development and nutrient absorption, thereby effectively improving the body’s immunity [102], which helps to reduce the incidence of diseases in adolescents and children. Piglet systemic and mucosal immunity is comparable to that of humans and is therefore commonly used to assess both innate and acquired immunity [103]. Oral administration of bovine colostrum significantly increased the concentrations of interleukin (IL)-2, interferon-γ, granulocyte-macrophage colony-stimulating factor and decreased the level of IL-6 in immunocompromised mice, suggesting that bovine colostrum can exhibit immunomodulatory effects by modulating spleen T lymphocyte subsets [104]. Studies have shown that adding a dose of bovine colostrum to the diet has a positive effect on the intestinal morphology and immune status of early-weaned pigs [105]. At the same time, BC supplementation significantly stimulated the CD21+/CD3+ cell population of the ileal Peyer’s patch (iPP) in the intestinal mucosal immune cells of weaned piglets, and stimulated the increase in Th1 and Th2 cytokines in different organs of weaned piglets, suggesting that BC helps to promote the production of mammalian immune cells [106] and protect weaned piglets from allergic (food) and infectious (pathogen) diseases [107]. Clinical studies have found that the total number of URTI infections decreased dramatically from 8.6 ± 5.1 at baseline, to 5.5 ± 1.2 after two months, and to 5.7 ± 1.6 after six months, with a significant reduction in the number of URTIs, diarrhea attacks, and hospitalizations in children with recurrent upper respiratory tract infections (URTI), indicating that BC is effective in preventing URTI and diarrhea [108]. Similarly, in preschool children, six weeks of BC supplementation has been shown to prevent and reduce the severity of URTIs in preschool children, and this protective effect can be sustained for up to 20 weeks with no significant side effects [109]. These results indicate that both long-term and short-term BC supplementation can provide better anti-URTI effects and higher safety in children. The defense mechanism of upper respiratory tract infection consists of two main points: the barrier formed by the mucosal epithelium effectively prevents microbial attack on tissues, and the immune protection provided by the body’s specific immune system [110,111]. Treatment with 20 g per day of BC for six weeks in regularly trained adolescents significantly enhanced their salivary immunoglobulin A (slgA) concentrations, suggesting that BC intervention may have a role in modulating mucosal immunity [112]. Therefore, it is reasonable to think that the reason for the anti-URTI protection provided by BC may be related to its increased mucosal regeneration of the upper respiratory tract. At the same time, in a double-blind randomized controlled trial, the use of BC in the treatment of acute diarrhea was effective, reducing the frequency and duration of diarrhea in children [113], presumably related to the high levels of immunoglobulin provided by BC. Another meta-analysis also showed a strong association between BC and a reduction in the frequency and symptoms of infectious diarrhea in children [114].

The above studies have shown that providing certain BC supplementation in children can significantly improve their immunity and reduce the incidence of diarrhea and URTI, which is related to the abundant immunoreactive components provided by BC, and the longer duration of this protective effect has a good application prospect.

### 6.3. Bovine Colostrum Helps Regulate Adult Immunity

BC has a significant modulating effect on adult immunity. In the athlete population, BC shows a particularly significant immunomodulatory effect. Studies have shown that prolonged or high-intensity training may lead to transient immune dysfunction, also known as immunosuppression, and strenuous exercise can also cause an increase in pro-inflammatory factors, leading to increased susceptibility to infection, and predisposition to respiratory tract infections and gastrointestinal diseases [115,116]. Bovine colostrum has a high concentration of IgA and IgG, which help to protect the mucosa and improve the resistance of the respiratory tract and gastrointestinal tract to infection [117,118,119]. Long-term supplementation with BC can significantly increase the concentration of IgG in athletes, improve the ability to resist infection, and effectively reduce the number of inflammatory markers in the athlete’s body [120]. Furthermore, BC supplementation during long-term training can increase serum IGF-I and salivary IgA concentrations in athletes [121]. However, a recent meta-analysis showed that BC supplementation had no significant effect on serum immunoglobulin, lymphocyte, and neutrophil concentrations, or IgA concentrations in saliva in athletes and manual laborers [122]; however, BC administration did have a positive effect on the health of athletes, possibly through other active ingredients in BC. At present, research on the immunomodulatory effect of BC in adults is mainly concentrated in the athlete population, but some studies have explored the immune-boosting effect in the non-athlete population. Results from a randomized, triple-blind, placebo-controlled trial showed that BC supplementation significantly reduced the risk of URTI and the severity of URTI in college students in the high-risk group [123]. After 21 days of daily intake of 45 g of bovine colostrum, there was an increase in the hemoglobin, serum albumin levels, and number of blood lymphocytes in adults after femoral fracture, and an improvement in the physical function of patients, showing the good immunomodulatory effect of BC [124].

BC can promote the number of immunoreactive factors and immune proteins in the serum of adults, effectively regulate the body’s immunity, help improve the anti-infection ability of the human respiratory tract and gastrointestinal tract, and improve the body’s exercise ability. However, although the improvement in physical function and athletic performance of athletes with BC supplementation has been observed in most of the studies focusing on athletes, the results of the test indicators show that there are still contradictory results, which suggests that further analysis in combination with animal models is needed in future studies, or multiple experimental groups are set up for discussion.

### 6.4. Bovine Colostrum Helps to Improve the Activity of Immune Cells in the Elderly

**Figure 3:**
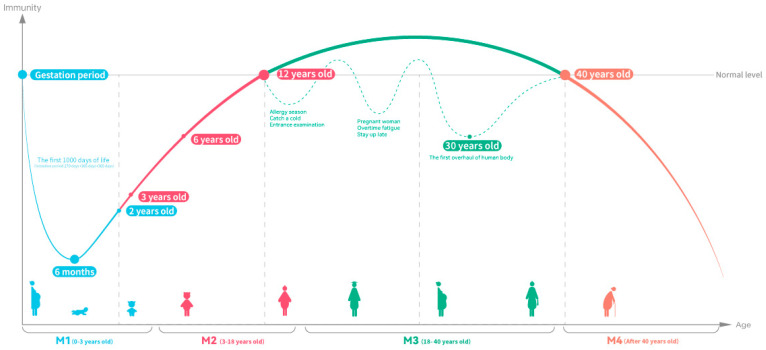
Atlas of immunity over a person’s lifetime (figure drawing is based on [79,80,81,100,132]).

The global population is ageing at an increasing rate, with the number of people over 65 expected to rise to 16% by 2050, compared to 10% in 2022 [125], and the increase in life expectancy has also led to an increase in the health of the elderly. Senescent cells in ageing populations are unable to respond quickly to immune protection when they are exposed to external adverse factors, making influenza more likely to occur [126]. Although healthy older adults have sufficient and well-functioning neutrophils, decreased antimicrobial activity and changes in oxygen metabolism have been observed in extremely elderly people [127]. Figure 3 summarizes the changes in immunity at various stages of the human process from birth to ageing, as well as the health needs in immunocompromised periods. In general, infants have poor immunity at 0~6 months of age and are completely dependent on breast milk or nutritional fortifiers to take in a large number of nutrients needed for immune construction, and the immune system begins to gradually improve after 6 months [80,81]. However, with increasing age, immunity begins to decline dramatically [128]. Therefore, maintaining stable immune function throughout the lifespan may help improve the health of the ageing population and save medical expenses in older populations, and the method of achieving recovery or stimulating immune enhancement through nutrition is generally accepted [129] because the process of immune response requires a large amount of energy support, and the strategy of achieving health through nutritional supplementation has been highly recognized [130,131].

Ageing is an inevitable physiological phenomenon in human beings, which brings about the ageing of cells and the decline of physiological functions. It is generally believed that there are two main types of senescence, namely, premature senescence of cells due to emergency injury, followed by replicational senescence caused by repeated cell cycles [133], and excessive accumulation of senescent cells, which increases the expression of pro-inflammatory factors [134], and although inflammation is a normal response of the body’s immune system to injury or infection, this response may last longer in older people, leading to the frequent occurrence of age-related diseases, chronic diseases, and tissue dysfunction [135,136,137]. The boost of immunity may help reduce the various diseases that can occur due to excessive inflammation and damage during ageing. Studies have shown that increased inflammation associated with ageing reduces IGF-1 activity, thereby affecting the anabolic and catabolic pathways of skeletal muscles [138]. BC, which is rich in IGF-1 with anti-inflammatory activity, has been shown to increase lower limb strength and reduce bone resorption in the elderly, providing a certain effect on improving bone health [139,140]. In a double-blind, randomized controlled trial of a 12-week BC intervention in older adults (aged 50 to 69 years), subsequent testing showed that regular consumption of skim milk with BC improved weight, blood pressure, and lower limb function, better controlled blood cholesterol levels, and potentially enhanced immune function in older adults [141]. Similarly, another clinical study also observed that the expression of various pro-inflammatory factors, such as CRP, IL-6 and TNF-α, was significantly reduced in the elderly after eating defatted BC, and the results of non-targeted metabolomic analysis showed that BC may cause changes in glycerophospholipid metabolism, cysteine, and methionine metabolism pathways, which effectively regulated the immune function and inflammatory status of the elderly. In summary, BC helps to reduce the release of inflammation and reduce the level of immunocompromise due to ageing in the elderly.

**Table 2:** Effect of colostrum on immunity in various age groups.

Subject	Dosages	Cyclicality	In the End	Ref.
≤34 weeks preterm	Slow transition from 10 mL/kg/day to full intake of 200 mL/kg/day	14 days	CD4+CD25+ FOXP3+ T lymphocytes % (FOXP3 Tregs) had higher levels at follow-up; sepsis severity and mortality tended to be lower in the BC group.	[96]
Premature pig	3~15 mL/kg/3 h	14 days	Body growth, intestinal hexose uptake and transit time were improved, and diarrhea and intestinal permeability were significantly reduced in the BC group of piglets, which also had lower densities of colonic mucosa-associated bacteria and some putative pathogens, as well as higher levels of intestinal villi, mucosal mass, brush border enzyme activity, and colonic short-chain fatty acids, compared to those consuming the formula.	[97]
Normal baby	20 mL/d	2 weeks	Infants who received daily BC containing human rotavirus antibodies did not develop rotavirus-induced diarrhea, whereas all infants who received BC intervention after the onset of symptoms developed diarrhea. Oral BC with human rotavirus antibodies may be an effective and safe method of preventing diarrhea caused by rotavirus infection.	[99]
Rotavirus-infected mice	50 μL/d	1~24 h	In a mouse model of human rotavirus infection, BC feeding containing antibodies to human rotavirus was effective in preventing gastroenteritis in mice.	[99]
URTI Children	For children under 2 years old 3 g per day, children over 2 years old 6 g per day	1~6 months	BC is effective in preventing recurrent URTIs and diarrhea and reducing the number of episodes and length of hospital stay due to infection.	[108]
Children aged 3 to 7 years	Dose 1.0 g/d for the first 15 days and 0.5 g/d for the next 30 days	6 weeks	BC supplementation in preschool children was well tolerated, safe, and prevented the frequency of URTIs and their severity, with effectiveness lasting up to 21 weeks.	[109]
adolescents	20 g/d	6 weeks	6 weeks of colostrum supplementation increases sIgA concentrations during adolescent training.	[112]
Children aged 2 to 6 years	/	48 h	BC is effective in the treatment of acute diarrhea and can be used as an adjunctive therapy as it reduces the frequency and duration of diarrhea.	[113]
Footballer	3.2 g/d	6 months	BC was able to reduce the expression of inflammatory factor TNF-α during athletes’ training, increase the number of immunoglobulins in the body China, and improve the resistance to infection and immunity.	[120]
Adults after femur fracture surgery	45 g	21 days	BC increases appetite and provides hemoglobin, serum albumin levels and blood lymphocyte counts, suggesting that BC accelerates weight gain and physical function after surgery	[124]
University student	Dose of 1.0 g/d for the first 15 days, 0.5 g/d for the next 30 days, then supplemented at 1.0 g/d for 7 d starting on day 87	45 + 7d	Supplementation with BC significantly reduced the incidence of URTI, reduced the severity of URTI symptoms, and did not show any side effects or intestinal discomfort.	[123]
Older people aged 50–69	15 g (contains 150 mg of IgG)	12 weeks	BC helps to improve weight management, blood pressure, blood cholesterol, verbal memory, lower limb function and potentially immune function in older adults.	[141]
Older people aged 50–69	15 g (contains 150 mg of IgG)	12 weeks	Skimmed milk from BC may help to reduce the expression levels of various pro-inflammatory mediators, such as CRP, IL-6, and TNF-α, and induce changes in glycerophospholipid metabolism, cysteine, and methionine metabolic pathways, which may improve immune function in the elderly.	[141]

## 7. Summary

Immunity is closely related to physical health, and this study found that BC supplementation in infancy can make up for the lack of immunodeficiency in preterm infants, reduce infant feeding intolerance, NEC and LOS, and help the intestinal tract quickly establish an immune barrier. BC supplementation during adolescence can help reduce diarrhea and upper respiratory tract infections. BC supplementation in adulthood improves the anti-infection ability of the human respiratory tract and gastrointestinal tract and the body’s exercise capacity. BC supplementation in the elderly can help reduce the release of inflammatory factors and reduce the low immunity caused by aging in the elderly. Although it can be seen that bovine colostrum has good application value at all stages of the population, it is difficult to ignore the low yield of colostrum, which accounts for about 0.5% of the annual milk production of dairy cows. Although the production of colostrum in most healthy productions far exceeds the demand of calves, there are still many problems in the collection, processing, and preservation of colostrum [5]. Recently, Melnik et al. [142] identified commercial milk consumption as a key risk factor for estrogen receptor-α α-positive (ER+) BCa. This suggests that whether it is commercial dairy products or bovine colostrum, its safe intake and intake cycle should be considered in the process of application, to better provide the necessary immunity needs for one’s health.

In conclusion, bovine colostrum is an ingredient that has a positive effect on immunity in people of all ages. Compared to other commercial dairy products, the limits of flavin colistin M1, pathogenic bacteria, antibiotics, and stimulants are more prominent. At the same time, while we see the advantages of bovine colostrum, we should also pay attention to its limitations in human application, because the composition of BC is different from that of human colostrum. Specifically, the main protein of BC is casein, while the main protein in breast milk is whey protein, especially α-whey protein, which together with ovalbumin is still the reference protein with the best biological value, especially for muscle. Therefore, BC cannot completely replace breast milk. When used as a fortifier for breast milk, its ingredients must be adapted to the needs of preterm infants and must be free of other potential hazards such as Bacillus cereus. At the same time, more clinical trials are needed to support BC before it can be used in preterm infants. In addition, lactose intolerance in people, β-lactoglobulin allergy, and many other factors mean that bovine colostrum cannot completely replace human colostrum or cow’s milk. How to overcome these limitations through effective scientific means is the key to whether bovine colostrum can be widely used.

## Figures and Tables

**Table 1:** Comparison of the nutritional composition of bovine colostrum with five other milk sources.

Ingredient	CHM	MHM	CPM	MPM	BC	BM
Energy (kcal/dL)	58	69	42.3	50.5	130	–
Fat (g/L)	20–50	32–38	12–26	24–35	50–80	37–39
Protein (g/L)	8–37	9.0–12.0	19–41	12.7–26	60–149	34–36
Carbohydrates (g/L)	–	76	22–62	30–77	–	–
Casein (g/L)	3.0–5.6	4–4.8	–	–	26–43	2–30
Whey (g/L)	4.3–11.1	6–7.2	–	–	35–120	4–50
Immunoglobulin (g/L)	1.14–20	1.2	–	–	42–90	0.4–1.0
Lactose (g/L)	44–72	50–78	22–49	28–73	18.9–32	49
IgG (g/L)	0.05–0.43	0.03–0.06	–	–	20–200	0.15–0.8
IgG1 (g/L)	–	–	–	–	15–180	0.3–0.6
IgG2 (g/L)	–	–	–	–	1–3	0.06–0.12
SIgA (g/L)	3.5–17.35	1.0–1.7	–	2.6	1.7–6.2	0.04–0.14
IgM (g/L)	0.15–1.59	0.03–0.10	–	–	3.7–9.0	0.03–0.10
α-Lactalbumin (g/L)	2.56	2–3	–	–	2.04	1–1.5
β-Lactoglobulin (g/L)	–	–	–	–	14.3	–
Lactoferrin (g/L)	5.05–7.0	1.0–2.7	–	–	0.8–5.0 ^a^	0.01–0.75
Whey protein (%)	–	–	–	–	6	0.4–0.5
Lactoperoxidas (mg/L)	5.17	5.17	–	–	11–45	13–30
Lysozyme (mg/L)	270–430	160–460	–	–	0.14–0.7	0.07–0.6
EGF (μg/L)	35–438	20–111	–	–	4–324.2	2–155
TGF-β (mg/L)	1.4–40	0.953	–	–	0.15–2.0	0.013–0.071
TGF-α (μg/L)	2.2–7.2	–	–	–	2.2–7.2	–
IGF (mg/L)	18	–	–	–	10	–

Data sources: [3,15,17,18,19,20,21,22,23,24,25,26,27,28,29,30,31,32,33,34,35]. Note: “–” indicates no reference; CHM = colostrum for healthy mothers; CPM = colostrum for premature mothers; MHM = mature milk of healthy mothers; MPM = mature milk for preterm mothers; BC = bovine colostrum; BM = bovine matured milk; TGF = transforming growth factor; EGF = epidermal growth factor; IGF = insulin-like growth factor. ^a^: Includes values before and after sterilization. The content of lactoferrin will decrease rapidly after high temperature sterilization, so reasonable sterilization methods are worth discussing and solving in the future.
